# The enhancer RNA ADCY10P1 is associated with the progression of ovarian cancer

**DOI:** 10.1186/s13048-022-00987-1

**Published:** 2022-05-14

**Authors:** Jiaya Mo, Lianghao Zhang, Huiqing Li, Haoran Duan, Dong Wang, Xiaolei Zhao, Ya Xie

**Affiliations:** 1 Department of Gynecology and Obstetrics, The First Affiliated Hospital of Zhengzhou University, Zhengzhou, Henan China; 2 Department of Urology, The First Affiliated Hospital of Zhengzhou University, Zhengzhou, Henan, China; 3 Department of Information, The First Affiliated Hospital of Zhengzhou University, Zhengzhou, Henan China

**Keywords:** Ovarian cancer, Enhancer RNAs, Prognosis, Proliferation, Metastasis

## Abstract

**Background:**

Emerging evidence identifies enhancer RNAs (eRNAs) as a class of regulatory ncRNAs that can contribute to the transcription of target genes. In this study, we used an integrated data analysis method to identify the important role of eRNAs in ovarian cancer (OC).

**Methods:**

Gene expression profiles and clinical information from The Cancer Genome Atlas (TCGA) database were used for this study. Based on expression analysis using GEPIA2 gene and Kaplan–Meier survival was performed to ensure the significance of the selected enhancer RNA ADCY10P1 in OC. Next, we explored the correlation and clinical significance between ADCY10P1 and target gene NFYA. Furthermore, we evaluated the effects of overexpression of ADCY10P1 on the proliferation, migration, invasion and epithelial-mesenchymal transformation (EMT) of OC cell lines. We also investigated the biological function enrichment score of ADCY10P1 and verified it with OC cell lines. Finally, external validation was conducted, and the prognostic value of the ADCY10P1 in different tumors was demonstrated.

**Results:**

We selected the eRNA ADCY10P1 associated with OC prognosis, with NFYA as its predicted target gene. Low ADCY10P1 expression was found to be associated with poor overall survival, high histological grade, and advanced stage of OC. Additionally, overexpression of ADCY10P1 inhibited the proliferation, migration, invasion and EMT phenotype of OC cell lines. Furthermore, ADCY10P1 was observed to inhibit glycolysis and fatty acid metabolism, thereby affecting OC progression. Meanwhile, OC tissue samples were externally validated. In addition, the pan-cancer analysis revealed that ADCY10P1 had prognostic value in other cancers.

**Conclusions:**

This study showed that ADCY10P1 plays a key role in OC progression and may facilitate prognosis prediction.

## Background

Ovarian cancer (OC) is the third most common type of cancer in women and is the leading cause of mortality in gynecological malignancies. In 2020, in the United States alone, approximately 21,750 new cases of OC were diagnosed, and 13,940 women died from the disease [1, 2]. The standard treatment for OC is cytoreductive surgery followed by chemotherapy. However, most patients develop chemotherapeutic resistance within six months of surgery [3]. The overall five-year survival rate for OC is only 47% due to diagnoses typically occurring at a late stage and a high recurrence rate [4]. Moreover, the measurement of CA125 concentrations, pelvic ultrasound, tumour grade, and histopathological classification have failed to provide an accurate prognosis prediction for patients with OC [5]. Therefore, the molecular biomarkers of OC still need to be explored.

Emerging evidence demonstrates that human RNA transcripts are mainly non-coding RNAs (ncRNAs) consisting of small interfering RNAs (siRNAs), microRNAs, and long non-coding RNAs (lncRNAs) [6]. Among the ncRNAs, lncRNAs are more than 200 nucleotides in length and participate in multiple biological processes, including gene expression, protein translation, histone methylation, RNA splicing, and microRNA regulation [7, 8]. Moreover, various studies have shown that lncRNAs may play a significant role in the initiation and progression of cancers [9]. Additionally, altered lncRNA expression has been reported as a biomarker for cancer therapeutic response and prognosis [10]. However, the functions and mechanisms of many cancer-associated lncRNAs are poorly understood.

Recent studies have identified enhancer RNAs (eRNAs) as a class of regulatory ncRNAs that can increase the transcription of target genes [11]. There have been numerous reports that eRNAs are realised by interacting with RNA polymerase II and a co-transcription complex to mediate promoter-enhancer looping [12]. Enhancers may also regulate target gene expression via transcripts produced from the enhancer regions themselves [13]. It is well known that eRNAs can regulate the maintenance of hundreds of cells. Hence, eRNA dysregulation plays a key role in disease progression and tumour development [14]. In colon cancer, the lncRNA colon cancer-associated transcript 1 is transcribed from this super-enhancer belonging to eRNA, which regulates c-MYC oncogene expression [15]. Furthermore, most expression and function of genes involved in cancer signalling pathways are linked to eRNAs [16]. However, the numerous eRNAs involved in OC progression and prognosis have rarely been reported.

Therefore, we aimed to explore the potential clinical utility of eRNAs with respect to their functions, mechanisms, association with OC prognosis, and potential therapeutic targets. This study found that the eRNA ADCY10P1 is strongly associated with overall survival (OS) and clinical features among patients with OC. Additionally, overexpression of ADCY10P1 inhibited the proliferation, migration, invasion and EMT of OC cell lines. Furthermore, when expressed at a high level, ADCY10P1 was found to act as a protective lncRNA that can inhibit glycolysis and fatty acid metabolism, crucial for the aggressive proliferation of cancer cells.

## Results

### ADCY10P1 is a key prognostic eRNA of OC

**Fig. 1:**
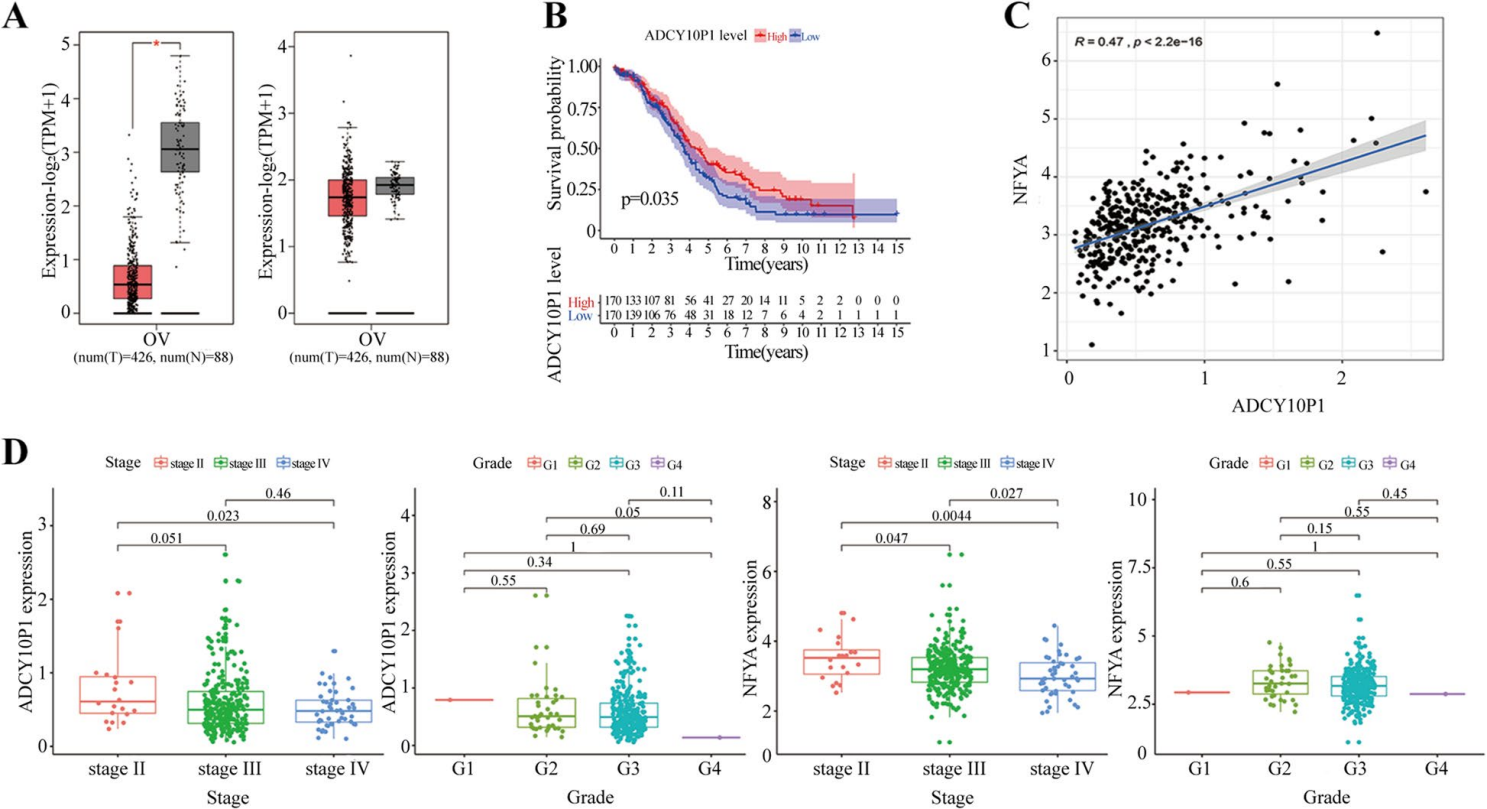
ADCY10P1 expression and clinical significance in ovarian cancer (OC). **A** ADCY10P1 and its predicted target gene NFYA median expression in OC tissues were significantly lower than normal tissues.**B** ADCY10P1 downregulation was significantly correlated with poorer overall survival (OS). **C** Correlation analysis between ADCY10P1 and NFYA. **D** ADCY10P1 expression decreased with advanced Federation of Gynecologists and Obstetrics (FIGO) stages

Our study identified ADCY10P1 as a putative eRNA associated with OC survival. From a total of 426 TCGA-OC samples and 88 normal ovarian samples, we found that the median expression of both ADCY10P1 and NFYA was lower in OC tissue samples than in normal tissue samples (Fig. 1A). A total of 340 OC samples with survival information are presented in Table 1. Low ADCY10P1 expression was associated with poor OS (Fig. 1B, Kaplan–Meier log-rank test, *P* = 0.035). Furthermore, ADCY10P1 expression was correlated with the predicted target gene NFYA expression (Fig. 1C, r = 0.47; *P* < 0.001). In addition, to further establish whether ADCY10P1 expression is involved in OC progression, our results showed it was negatively correlated with Federation of Gynecologists and Obstetrics (FIGO) stage (Fig. 1D, P < 0.05). These results suggested that ADCY10P1, as a key eRNA plays an important role in OC prognosis.

**Table 1.** Summary of clinical characteristics of TCGA-OV patient data sets

Characteristic	Type	TCGA-OV data set (*n* = 340)
Vital status, n (%)	Alive	140(41.2)
	Dead	200(58.8)
Age, n (%)	< 60	176(51.8)
	≥ 60	164(48.2)
FIGO-Stage, n (%)	Stage II	20(5.9)
	Stage III	269(79.1)
	Stage IV	48(14.1)
	Unknow	3(0.9)
Grade, n (%)	G1	1(0.3)
	G2	39(11.5)
	G3	292(85.9)
	G4	1(0.3)
	unknow	7(2.0)

### ADCY10P1 inhibits cell proliferation of OC

**Fig. 2:**
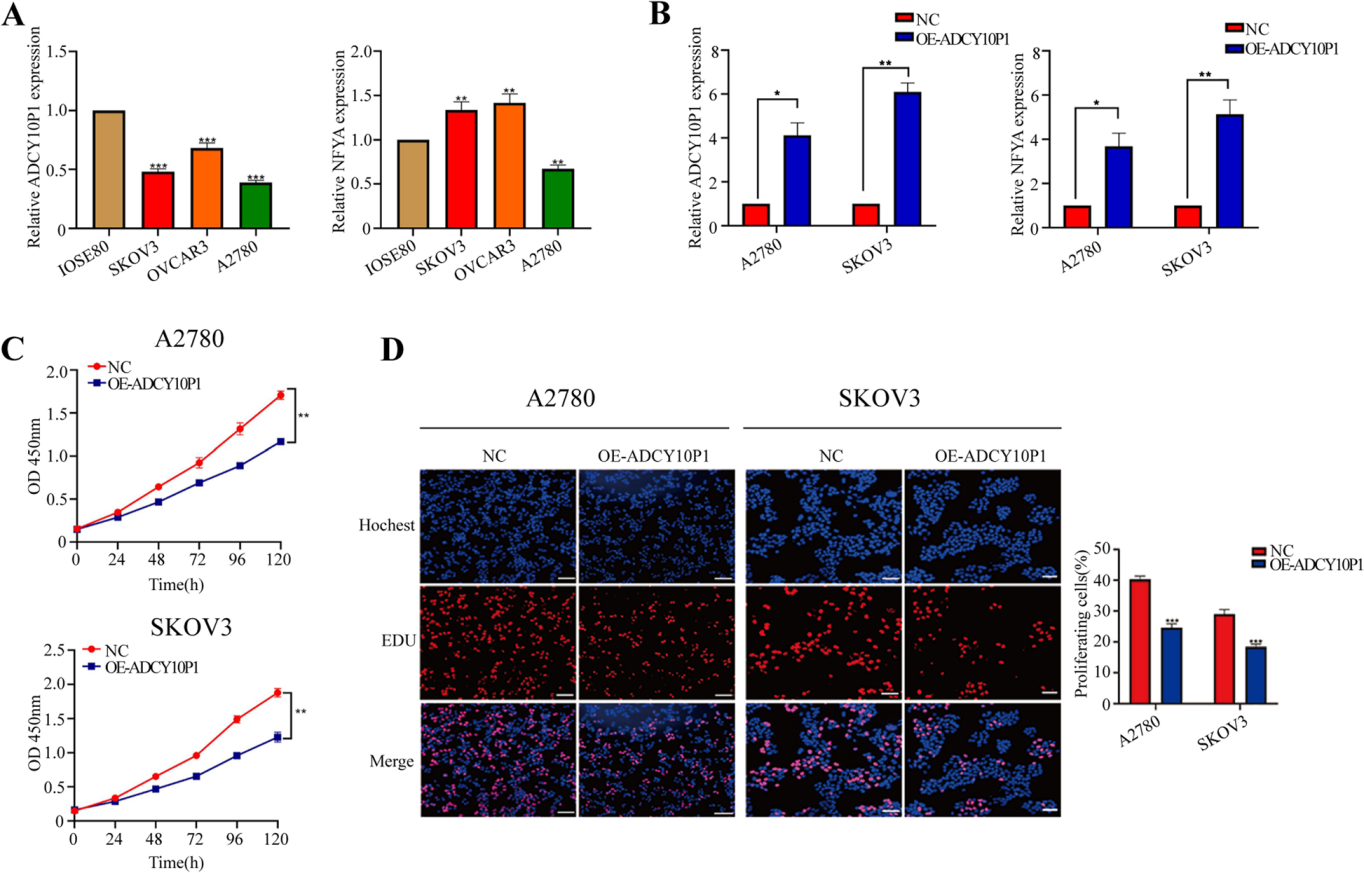
ADCY10P1 inhibits cell growth in ovarian cancer (OC) cells. **A** ADCY10P1 and NFYA expressions in IOSE80 cell line and OC cell lines were analysed by qRT-PCR. **B** ADCY10P1 overexpressing vector increased ADCY10P1 and NFYA expression in OC cells. **C** Cell growth viability was analysed by CCK-8 assay. **D** Overexpression of ADCY10P1 suppressed ovarian cancer cell proliferation by EdU assay.

Based on the above results, we hypothesized that ADCY10P1 could suppress OC progression. We first examined the expression levels of ADCY10P1 in three OC cell lines (A2780, SKOV3, OVCAR3) and normal ovary cell line (IOSE80) using qRT-PCR. The expression levels of ADCY10P1 were significantly lower in the OC cell lines compared with the IOSE80 cell line, while NFYA expression levels were higher in SKOV3 and OVCAR3 cell lines than in the IOSE80 cell line (Fig. 2A). Subsequently, we selected A2780 and SKOV3 cell lines for transfection with an overexpression ADCY10P1 plasmid. As confirmed by qRT-PCR, ADCY10P1 and NFYA expression were upregulated (Fig. 2B). We conducted CCK-8 and EdU assays to determine the proliferation changes in OC cell lines and investigate whether ADCY10P1 regulates OC cell proliferation. As shown in Fig. 2C-D, overexpression of ADCY10P1 significantly decreased OC cell lines proliferation. Therefore, these results indicated that ADCY10P1 inhibited OC cell proliferation.

### ADCY10P1 inhibits cell migration, invasion, and EMT of OC

**Fig. 3:**
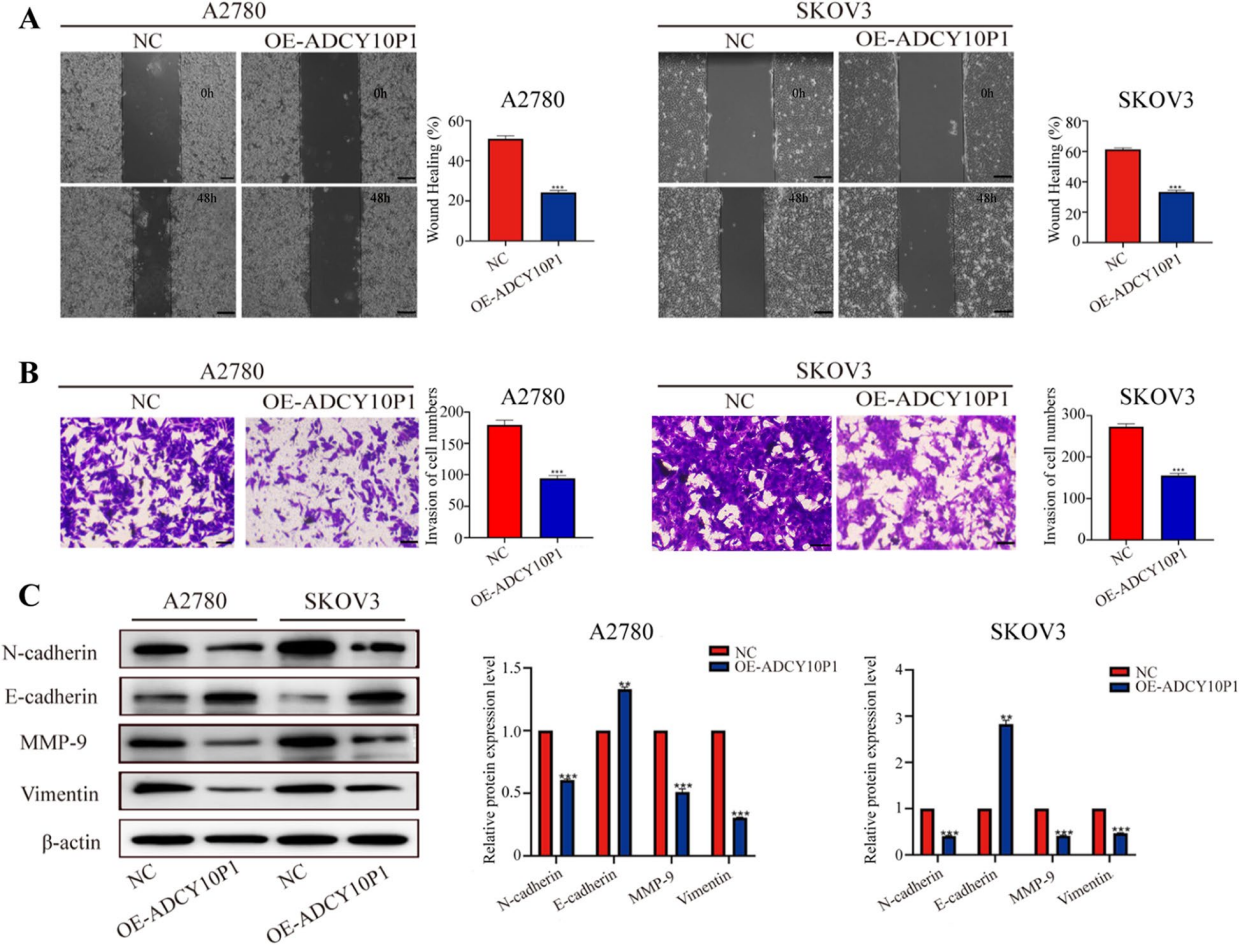
ADCY10P1 inhibits cell migration, invasion, and epithelial-mesenchymal transition (EMT) of ovarian cancer (OC) cells. **A** Changes in OC cells migration were measured using the wound-healing assay. **B** Transwell assay showed that overexpression of ADCY10P1 inhibited OC cells invasion. **C** The expression of EMT markers was determined using western blot assay

We used wound-healing and transwell assays to determine whether ADCY10P1 regulated the migration and invasion ability of OC cells. We found that OC cells overexpression of ADCY10P1 migrated at a slower rate than control cells (Fig. 3A). Similarly, the transwell assay results showed that control cells had a stronger invasion ability than OC cells with overexpressed ADCY10P1 (Fig. 3B). We then performed western blotting to evaluate the effect of ADCY10P1 on OC cell EMT. The results showed that overexpression of ADCY10P1 decreased N-cadherin, matrix metallopeptidase (MMP)-9 and Vimentin expression but increased E-cadherin expression (Fig. 3C). These results indicated that ADCY10P1 affects migration, invasion, and EMT of OC cells.

### ADCY10P1 inhibits glycolysis and fatty acid metabolism via deregulation of glycolysis and lipogenic enzymes

**Fig. 4:**
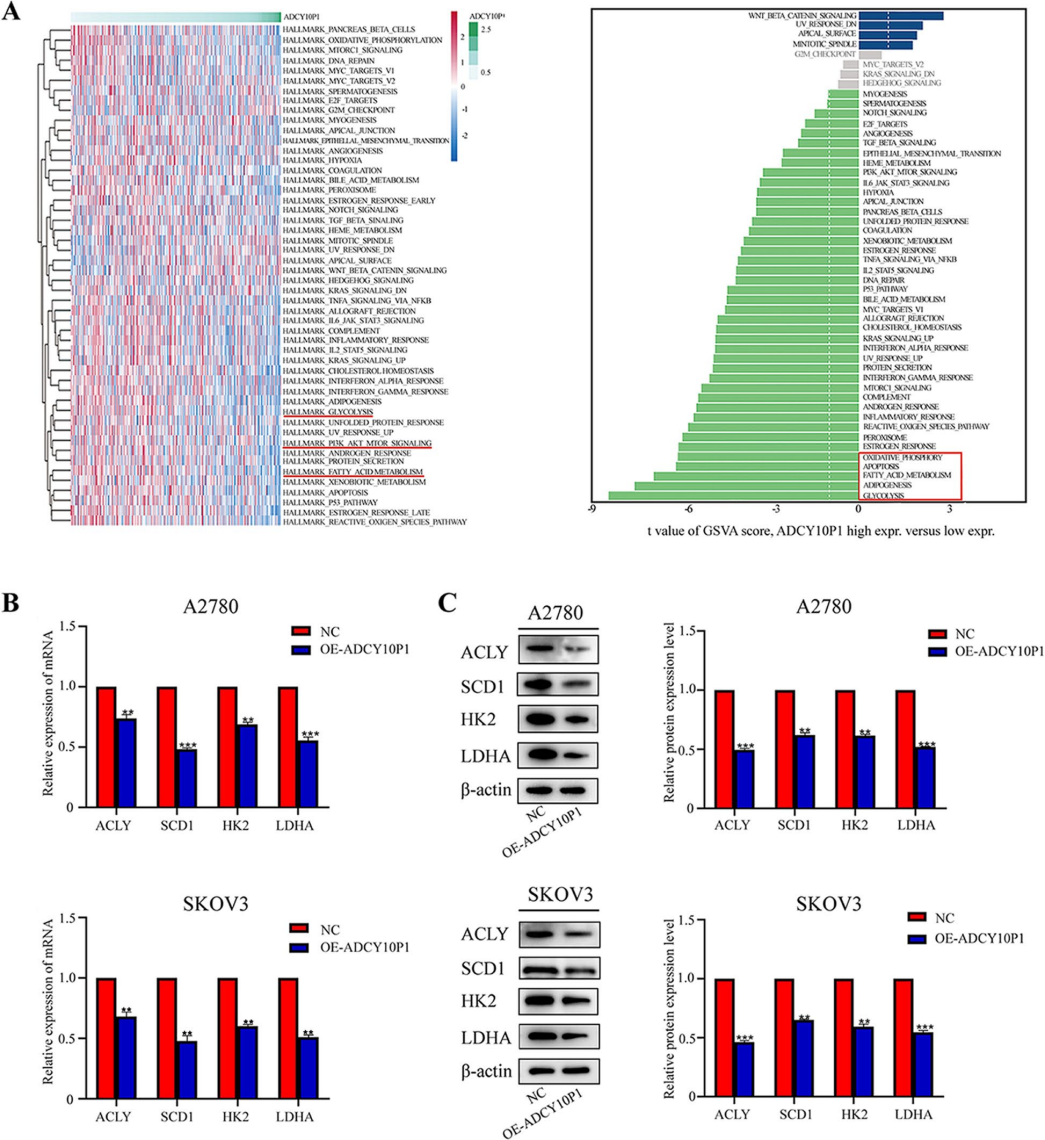
ADCY10P1 inhibits glycolysis and fatty acid metabolism of ovarian cancer (OC) cells. **A** Biological function analysis of ADCY10P1. **B** qRT-PCR showed that overexpression of ADCY10P1 inhibited glycolysis enzymes and fatty acid metabolism enzymes. **C** Glycolysis enzymes and fatty acid metabolism enzymes were examined using western blot assay

In recent years, glycolysis and fatty acid metabolism have provided energy for the high metabolic demand of tumour cells. Moreover, increasing evidence shows that glycolysis and fatty acid metabolism play important roles in tumour development and progression [17, 18]. As a result of the biological function of ADCY10P1, the heatmap indicated that low ADCY10P1 expression was enriched in fatty acid metabolism, PI3K/AKT signalling and glycolysis metabolism pathways. Meanwhile, as per the GSVA bar plot, low ADCY10P1 expression was found to be highly associated with glycolysis, adipogenesis, fatty acid metabolism, apoptosis, and oxidative phosphorylation pathways (Fig. 4A). We also examined the expression of glycolysis enzymes (hexokinases 2 [HK2], and lactate dehydrogenase A [LDHA]) and fatty acid metabolism enzymes (ATP citrate lyase [ACLY], and stearoyl CoA desaturase 1 [SCD1]). The results showed that the expression levels of HK2, LDHA, ACLY and SCD1 were decreased in cells with overexpressed ADCY10P1 (Fig. 4 B-C). In conclusion, ADCY0P1 inhibits glycolysis and fatty acid metabolism.

### Prognostic value of ADCY10P1 in different types of cancers

**Fig. 5:**
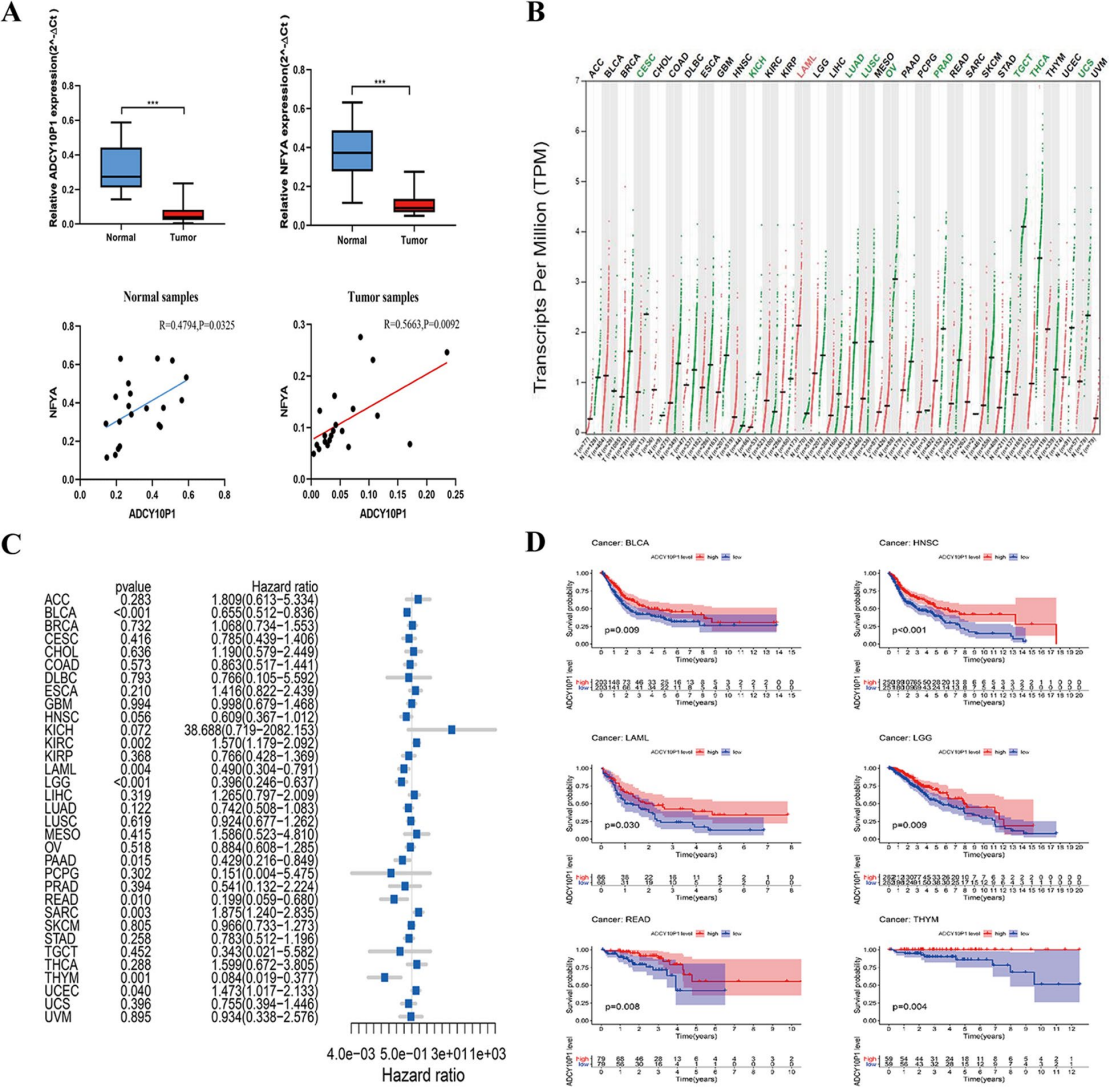
Identifying the prognostic effect of ADCY10P1 in pan-cancer. **A** Validated expression and correlation of ADCY10P1 and NFYA in ovarian cancer (OC) tissues. **B** ADCY10P1 expression in pan-cancer from GEPIA2 database. **C** Correlation of ADCY10P1 expression and hazard ratio in pan-cancer. **D** ADCY10P1 expression downregulation was significantly correlated with poorer overall survival (OS) in bladder urothelial carcinoma (BLCA), head and neck squamous cell carcinoma (HNSC), acute myeloid leukaemia (LAML), brain lower-grade glioma (LGG), rectum adenocarcinoma (READ), and thymoma (THYM)

We used qRT-PCR to measure the relative expression levels of ADCY10P1 and NFYA in 20 patients with OC to further validate ADCY10P1 expression in OC tissue samples. Comparison of matched normal ovarian and OC samples indicated that ADCY10P1 and NFYA expression levels were significantly downregulated in OC samples (Fig. 5A, P < 0.001). In addition, there was a significantly positive correlation between the expression levels of ADCY10P1 and NFYA in normal ovarian samples (*r* = 0.4794, *P* = 0.0325) and OC samples (*r* = 0.5663, *P* = 0.0092). In addition, we found that ADCY10P1 was more highly expressed in normal tissues than in tumour tissues, such as in cervical and endocervical cancers (CESC), kidney chromophobe (KICH), and lung adenocarcinoma (Fig. 5B). Moreover, the prognostic effect of ADCY10P1 in other cancer has also been investigated. ADCY10P1 also plays important prognostic roles in bladder urothelial carcinoma (BLCA), head and neck squamous cell carcinoma (HNSC), acute myeloid leukemia (LAML), brain lower-grade glioma (LGG), rectum adenocarcinoma (READ), and thymoma (THYM) (Fig. 5C-D). These results showed that ADCY10P1 might be a prognostic biomarker in OC and other types of cancers.

## Discussion

Despite new advances in treatment, including the introduction of targeted therapy, biotherapy, and immunotherapy, OC continues to have the highest mortality rate among women with reproductive system tumours, with an overall 5-year survival rate of 47% [4]. Therefore, it is important to explore clinical prognostic markers and understand the molecular changes and mechanisms associated with OC to achieve early diagnosis.

Enhancer RNAs as a kind of nRNAs are transcribed from enhancer elements [19]. Recently, a large amount of evidence has shown that eRNAs can regulate the occurrence and development of tumours [20]. For example, lncRNA FAL1 can promote cell proliferation, invasion, migration, and lower G0/G1 arrest in non-small cell lung cancer (NSCLC) cells [21]. In vitro knockdown of eRNA SMAD7 can suppresse tumorigenesis and progression of bladder cancer by CRISPR-Cas13a [22]. Enhancer RNA LINC02257 was highly expressed in colorectal cancer tissues and was associated with unfavorable prognosis [23]. However, studies showing the association between eRNAs and OC are comparatively less.

This study obtained RNA expression profile data and clinical information from the TCGA cohort to analyse the correlation between eRNAs and OC prognosis. We detected that the eRNA ADCY10P1 could be a prognostic biomarker of OC. Clinical correlation analysis found low expression levels of ADCY10P1 to higher histological grade, advanced stage, and poor prognosis. Meanwhile, we identified that ADCY10P1 is located in the enhancer region of NFYA and plays a positive role in regulating its expression. Increasing evidence has shown that NFYA plays a key role in various cancers, including lung, breast, and gastric cancer [24–26]. It can bind to CCAAT sites, which increases the expression of pro-growth genes [27]. However, the median expression of NFYA was found to decrease in OC tissues. Our study also found that ADCY10P1 and NFYA were highly expressed in normal ovarian tissues, while their expressions were decreased in OC tissues. Furthermore, ADCY10P1 was positively correlated with NFYA in normal ovarian tissues and OC tissues.

ADCY10P1 is an emerging cancer-related biomarker whose mechanism needs to be explored. The biological result revealed that low ADCY10P1 expression might drive tumour proliferation and invasion by affecting glycolysis, adipogenesis and fatty acid metabolism. Glycolysis and fatty acid metabolism have long been considered the major metabolic processes involved in energy production in cancer cells. Previously, reprogramming energy metabolism was confirmed to modulate tumour progression and metastasis [28, 29]. Our results showed that overexpression of ADCY10P1 inhibited the proliferation, migration, invasion, EMT, glycolysis enzymes, and fatty acid metabolism enzymes in OC cells. Thus, we speculated that overexpression of ADCY10P1 may regulate glycolysis and fatty acid metabolism to suppress OC progression.

Moreover, we investigated the role of ADCY10P1 in different cancers. The results revealed that ADCY10P1 plays a role as tumour suppressor in OC. A similar result was obtained, this study provides the first evidence that ADCY10P1 could be a novel prognostic biomarker for BLCA, HNSC, LAML, LGG, READ, and THYM. However, our study had some limitations. This predictive model was based on the TCGA database, which needed a larger clinical cohort and more samples to assess the role of prognostic ability further. Moreover, the potential function of ADCY10P1 also requires further investigation.

## Conclusion

In conclusion, the eRNA of ADCY10P1 can be used as a prognostic biomarker in OC. In addition, ADCY10P1 acts as a tumour suppressor and inhibits the proliferation, migration, invasion, EMT, glycolysis and fatty acid metabolism of OC cells.

## Materials and methods

### Clinical sample collection and cell cultures

All tissue samples were obtained from patients treated at The First Affiliated Hospital of Zhengzhou University. Each patient signed an informed consent form, and this study was approved by the First Affiliated Hospital of Zhengzhou University Institutional Review Board. Tissues samples were collected from OC patients with unilateral ovarian invasion and normal contralateral ovary, including 17 cases of high-grade serous carcinoma, 2 cases of mucinous cystadenoma and 1 case of mucinous carcinoma. Human OC cell lines (A2780, SKOV3, OVCAR3) and a normal ovary cell line (IOSE80) were purchased from the Cell Bank of Chinese Academy of Sciences (Shanghai, China). All cell lines were authenticated using the short tandem repeat (STR) profiling test. Then, IOSE80, A2780, OVCAR3 cells were cultured in RPMI-1640 Medium (Biological Industries, USA) supplemented with 10% fetal bovine serum (FBS, Biological Industries, USA), 100 units/ml penicillin, and 100 μg/ml streptomycin (Biological Industries, USA). SKOV3 cell was maintained in McCoy’s 5A Medium (Biological Industries, USA) supplemented with 10% FBS, 100 U/ml penicillin, and 100 μg/ml streptomycin.

### Extracting and identifying of prognostic eRNAs in OC through data analysis

The gene expression profiles for OC and 33 other types of cancers were downloaded from the TCGA database (https://cancergenome.nih.gov/). The clinical data of patients with OC, including survival time, age, survival state, survival grade, and gene expression, were obtained from TCGA. Using these data, we explored the association between putative eRNAs and OS in patients with OC using the “survival” and “survminer” packages of the R software (v4.0.0: http://www.r-project.org) (*P* < 0.05). Co-expression analysis was performed between the putative eRNAs and their predicted targets. Furthermore, we identified the expression levels of the eRNAs and their predicted targets in OC and other types of cancers based on TCGA and Genotype-Tissue Expression (GTEx) data from the gene expression profiling interactive analysis (GEPIA) 2 database (http://gepia2.cancer-pku.cn), [30]. Subsequently, we chose the eRNA consistent with survival-associated and tumour sample expression for further analysis. Next, the relationship between the expression of the selected eRNA and the clinicopathological characteristics of OC was examined. Finally, we used 20 pairs of matched OC and normal ovarian tissues for further verification using qRT-PCR. In addition, we verified the OS of the selected eRNA in the 33 other types of cancers.

### Gene bioinformatics enrichment analysis

Gene set variation analysis (GSVA) was performed using the default parameters of the GSVA package in R to explore the relationship between the selected eRNA and the biological function enrichment score [31]. Additionally, the "Hallmark" gene was downloaded from the Molecular Signatures Database (http://software.broadinstitute.org/gsea/index.jsp). The result was obtained using the “pheatmap”package in the R software.

### RNA extraction and quantitative real-time PCR

Total RNA was extracted from tissue samples or cell lines using TRIzol™ reagent (Thermo Fisher Scientific, Waltham, MA, USA) to synthesise cDNAs using the PrimeScript RT reagent kit (Takara Bio, Inc., Beijing, China) according to the manufacturer’s instructions. Quantitative real-time PCR analysis was performed using the SYBR Green PCR kit (Takara Bio, Inc., Beijing, China). The primer sequences are shown as follows: ADCY10P1 (forward: 5'-CACCCTGACCTACAAGTCGGAAC-3'; reverse: 5'- CTACAACCTGCCCAATCACCAA-3'), NFYA (forward: 5'-CAGTGGAGGCCAGCTAATCAC-3'; reverse: 5'-CCAGGTGGGACCAACTGTATT-3'), ACLY (forward: 5'-GAAGGGAGTGACCATCATCG-3'; reverse: 5'- TTAAAGCACCCAGGCTTGAT-3'), SCD1 (forward: 5'-AGAATGGAGGAGATAAGT-3'; reverse: 5'-TAGCAGAGACATAAGGAT-3'), HK2 (forward: 5'-AAGGCTTCAAG GCATCTG-3'; reverse: 5'-GCCAGGTCCTTCACTGTCTC-3'), LDHA (forward: 5'- ATGGCAACTCTAAAGGATCA-3'; reverse: 5'- GCAACTTGCAGTTCGGGC-3') and GADPH (forward: 5'-ATGACATCAAGAAGGTGGTG-3'; reverse: 5'-CATACCAGGAAATGAGCTTG-3'). The relative expression of ADCY10P1 and NFYA were calculated by 2^−∆ct^ or 2^−∆∆ct^ method.

### Cell transfection

The pLV3-CMV-ADCY10P1 was used to construct the plasmid expressing ADCY10P1in mammalian cells. Then, the plasmid was transfected in A2780 and SKOV3 cells using Lipofectamine 3000 (Invitrogen, USA) to overexpress ADCY10P1. Meanwhile, an empty plasmid was used as a negative control.

### Cell proliferation assay

Cell growth was assessed using the Cell Counting Kit-8 (CCK8) and Ethynyl-2-deoxyuridine (EdU). A2780 cells (3× 10^3^ cells/ well) or SKOV3 cells (3× 10^3^ cells/well) were seeded into a 96-well plate with 100 µL of complete medium. After grown at 37 °C in a humidified incubator containing 5% CO_2_ for 0, 24, 48, 72, 96 and 120 h, 10 μL CCK-8 solution (MedChemExpress, New Jersey, USA) was added, and the cells were incubated for 3 h in a dark cell incubator at 37 °C with 5% CO_2_. The absorbance was measured at 450 nm using a microplate reader. We used an EdU assay kit (Meilunbio, Dalian, China) to measured OC cell proliferation, following the manufacturer’s protocol. Finally, fluorescence microscopy (Olympus, Tokyo, Japan) was used to visualized cell fluorescence.

### Cell migration and invasion assays

For wound-healing assays, OC cells were seeded into 6-well cell culture plates, grown to 90% confluence, and scratch wounds were created with a sterile 10 μL plastic pipette tip. The cells were cultured in a medium with 1% FBS. The scratches were photographed at 0 h and 48 h. The relative migration area (%) was measured using Image J software. For OC cell invasion assays, 200 μL cell suspension without FBS was seeded in transwell upper chambers coated with Matrigel (Corning, USA), and medium with 20% FBS was added into the lower chambers. After incubation for 48 h, the invasive cells were fixed with methanol, stained with 0.1% crystal violet and imaged.

### Western blot assay

Cells were lysed with radioimmunoprecipitation (RIPA) buffer containing phenylmethylsulfonyl fluoride (PMSF) (Solarbio, Beijing, China) and PhosSTOP™ (Merck, Darmstadt, Germany). Proteins were separated by sodium dodecyl sulphate–polyacrylamide gel electrophoresis (SDS–PAGE) and transferred to polyvinylidene fluoride (PVDF) membranes. The PVDF membranes were blocked with TBST containing 5% skim milk and incubated overnight at 4 °C with the MMP9 (NO. 13667), HK2 (NO. 2867) and LDHA (NO. 3582) antibodies from Cell Signaling Technology (MA, USA), and N-cadherin (NO. AF4039), vimentin (NO. AF7013) and SCD1(NO. DF13253) antibodies from Affinity Biosciences (OH, USA), and E-cadherin (NO. 20874–1-AP) and ACLY (NO. 15421–2-AP) antibodies from Proteintech (Wuhan, China). Thereafter, the membranes were incubated with the horseradish peroxidase (HRP)-conjugated secondary antibody at room temperature for 1 h, and the signals were visualized with an enhanced chemiluminescence ECL kit (Meilunbio, Dalian, China).

### Statistics analysis

Statistical analyses were carried out using SPSS 22.0 (IBM, Chicago, IL, USA). Differences between groups were assessed using Chi-square test, paired sample *t*-test, Student’s *t*-test, or One-way ANOVA as appropriate. Additionally, the correlation strength was evaluated using Pearson’s correlation coefficient (r). Statistical significance was set at *P* < 0.05, and two-tailed P values were assumed.

## Data Availability

The datasets and materials used and analyzed during the current study are available from the corresponding author on reasonable request.

## References

[1] Lheureux S, Braunstein M, Oza AM (2019). Epithelial ovarian cancer: Evolution of management in the era of precision medicine. CA Cancer J Clin.

[2] Siegel RL, Miller KD, Jemal A (2020). Cancer statistics, 2020. CA Cancer J Clin.

[3] Narod S (2016). Can advanced-stage ovarian cancer be cured?. Nat Rev Clin Oncol.

[4] de Almeida MMFM, Nagashima JB, Venzac B, Le GS, Songsasen N (2020). A dog oviduct-on-a-chip model of serous tubal intraepithelial carcinoma. Sci Rep.

[5] May T, Stewart JM, Bernardini MQ, Ferguson SE, Laframboise S, Jiang H, Rosen B (2018). The prognostic value of perioperative, pre-systemic therapy CA125 levels in patients with high-grade serous ovarian cancer. Int J Gynaecol Obstet.

[6] Xu Sh, Kong D, Chen Q, Ping Y, Pang D (2017). Oncogenic long noncoding RNA landscape in breast cancer. Mol Cancer.

[7] Kasprzak WK, Ahmed NA, Shapiro BA (2020). Modeling ligand docking to RNA in the design of RNA-based nanostructures. Curr Opin Biotechnol.

[8] Chen YG, Satpathy AT, Chang HY (2017). Gene regulation in the immune system by long noncoding RNAs. Nat Immunol.

[9] Gibb EA, Brown Carolyn J, Lam WL (2011). The functional role of long non-coding RNA in human carcinomas. Mol Cancer.

[10] Yang K, Hou Y, Li LZ, Wang W, Xie H, Rong Z, Lou G, Li K (2017). Identification of a six-lncRNA signature associated with recurrence of ovarian cancer. Sci Rep.

[11] Sartorelli V, Lauberth SM (2020). Enhancer RNAs are an important regulatory layer of the epigenome. Nat Struct Mol Biol.

[12] Lam MTY, Cho H, Lesch HP, Gosselin D, Heinz S, Tanaka-Oishi Y, Benner C, Kaikkonen MU, Kim AS, Kosaka M, Watt LCY, A, Grossman TR, Rosenfeld MG, Evans RM, Glass CK.  (2013). Rev-Erbs repress macrophage gene expression by inhibiting enhancer-directed transcription. Nature.

[13] Li W, Notani D, Ma Q, Tanasa B, Nunez E, Chen AY, Merkurjev D, Zhang J, Ohgi K, Song X, Oh S, Kim HS, Glass CK, Rosenfeld MG (2013). Functional roles of enhancer RNAs for oestrogen-dependent transcriptional activation. Nature.

[14] Rahnamoun H, Lu H, Duttke SH, Benner C, Glass CK, Lauberth SM (2017). Mutant p53 shapes the enhancer landscape of cancer cells in response to chronic immune signaling. Nat Commun.

[15] McCleland ML, Mesh K, Lorenzana E, Chopra VS, Segal E, Watanabe C, Haley B, Mayba O, Yaylaoglu M, Gnad F, Firestein R (2016). CCAT1 is an enhancer-templated RNA that predicts BET sensitivity in colorectal cancer. J Clin Invest.

[16] Zhang Z, Lee JH, Ruan H, Ye Y, Krakowiak J, Hu Q, Xiang Y, Gong J, Zhou B, Wang L, Lin C, Diao L, Mills GB, Li W, Han L (2019). Transcriptional landscape and clinical utility of enhancer RNAs for eRNA-targeted therapy in cancer. Nat Commun.

[17] Snaebjornsson MT, Janaki-Raman S, Schulze A (2020). Greasing the Wheels of the Cancer Machine: The Role of Lipid Metabolism in Cancer. [J]. Cell Metab.

[18] Li ZH, Peng YX, Li JX,Chen ZJ, Chen F, Tu J, Lin SB, Wang HS. 2020. N-methyladenosine regulates glycolysis of cancer cells through PDK4. [J]. Nat Commun, 11: 2578. 10.1038/s41467-020-16306-5 PMC7244544 32444598

[19] Adhikary S, Roy S, Chacon J, Gadad SS, Das C. 2021. Implications of enhancer transcription and eRNAs in cancer. [J]. Cancer Res. 2021;81(16):4174–82. 10.1158/0008-5472.CAN-20-4010 34016622

[20] Jiao W, Chen Y, Song H, Li D, Mei H, Yang F, Fang E, Wang X, Huang K, Zheng L, Tong Q (2018). HPSE enhancer RNA promotes cancer progression through driving chromatin looping and regulating hnRNPU/p300/EGR1/HPSE axis. Oncogene.

[21] Pan CF, Yao GL, Liu B, Ma T, Xia Y, Wei K, Wang J, Xu J, Chen L, Chen YJ (2017). Long Noncoding RNA FAL1 Promotes Cell Proliferation, Invasion and Epithelial-Mesenchymal Transition Through the PTEN/AKT Signaling Axis in Non-Small Cell Lung Cancer. Cell Physiol Biochem.

[22] Che WN, Ye ST, Cai AX, Cui XJ, Sun YD (2020). in vitro CRISPR-Cas13a Targeting the Enhancer RNA-SMAD7e Inhibits Bladder Cancer Development Both and. Front Mol Biosci.

[23] Xiao JB, Liu YJ, Yi J, Liu XW. 2021. LINC02257, an Enhancer RNA of Prognostic Value in Colon Adenocarcinoma, Correlates With Multi-Omics Immunotherapy-Related Analysis in 33 Cancers. [J]. Front Mol Biosci, 8: 646786. 10.3389/fmolb.2021.646786 PMC8121256 33996902

[24] Chatterjee B, Ghosh K, Suresh L, Kanade SR (2019). Curcumin ameliorates PRMT5-MEP50 arginine methyltransferase expression by decreasing the Sp1 and NF-YA transcription factors in the A549 and MCF-7 cells. [J]. Mol Cell Biochem.

[25] Dolfini D, Andrioletti V, Mantovani R (2019). Overexpression and alternative splicing of NF-YA in breast cancer. Sci Rep.

[26] Cao B, Zhao Y, Zhang Z, Li H, Xing J, Guo S, Qiu X, Zhang S, Min L, Zhu S (2018). Gene regulatory network construction identified NFYA as a diffuse subtype-specific prognostic factor in gastric cancer. Int J Oncol.

[27] Dolfini D, Andrioletti V, Mantovani R (2019). Overexpression and alternative splicing of NF-YA in breast cancer. Sci Rep.

[28] Georgakopoulos-Soares I, Chartoumpekis DV, Kyriazopoulou V, Zaravinos A (2020). EMT Factors and Metabolic Pathways in Cancer. Front Oncol.

[29] Luo XQ, Zheng EZ, Wei L, Zeng H, Qin H, Zhang XY, Liao M, Chen L, Zhao L, Ruan XZ, Yang P, Chen YX (2021). The fatty acid receptor CD36 promotes HCC progression through activating Src/PI3K/AKT axis-dependent aerobic glycolysis. Cell Death Dis.

[30] Tang Z, Kang B, Li C, Chen T, Zhang Z (2019). GEPIA2: an enhanced web server for large-scale expression profiling and interactive analysis. [J]. Nucleic Acids Res.

[31] Barkal AA, Brewer RE, Markovic M, Kowarsky M, Barkal SA, Zaro BW, Krishnan V, Hatakeyama J, Dorigo O, Barkal LJ, Weissman IL (2019). CD24 signalling through macrophage Siglec-10 is a target for cancer immunotherapy. Nature.

